# Novel compound heterozygous *TARS2* variants in a Chinese family with mitochondrial encephalomyopathy: a case report

**DOI:** 10.1186/s12881-020-01149-0

**Published:** 2020-11-05

**Authors:** Xiaojing Li, Bingwei Peng, Chi Hou, Jinliang Li, Yiru Zeng, Wenxiao Wu, Yinting Liao, Yang Tian, Wen-Xiong Chen

**Affiliations:** Department of Neurology, Guangdong Province, Guangzhou Women and Children’s Medical Center, Guangzhou Medical University, 9# Jin Sui Road, 510623 Guangzhou, People’s Republic of China

**Keywords:** Mitochondrial diseases, Encephalomyopathy, *TARS2*, Whole genome sequencing, Case report

## Abstract

**Background:**

Mitochondrial encephalomyopathy caused by bi-allelic deleterious variants in *TARS2* is rare. To date, only two pedigrees were reported in the literature and the connection between the gene and disease needs further study.

**Case presentation:**

We report one infant who presented with limb hypertonia, epilepsy, developmental delay, and increased serum lactate from a non-consanguineous Chinese family. Whole-genome sequencing was performed to help to underlie the cause. We identified compound heterozygous variants c.470C > G, p.Thr157Arg and c.2143G > A, p.Glu715Lys in *TARS2* and the variants were confirmed by Sanger sequencing. The patient was diagnosed with combined oxidative phosphorylation deficiency 21 according to the Online Mendelian Inheritance in Man (OMIM) database based on the clinical data and the deleterious effect of the two variants in *TARS2* predicted by in silico tools.

**Conclusions:**

We presented one case diagnosed with combined oxidative phosphorylation deficiency 21 based on clinical characteristics and genetic analysis. This is the first case in China and the fourth case in the world based on our document retrieval. This study facilitates the understanding of combined oxidative phosphorylation deficiency disease and demonstrates that the next-generation sequencing has a high potential to study inherited disease with high phenotypic heterogeneity and genetic heterogeneity including mitochondrial diseases such as combined oxidative phosphorylation deficiency.

## Background

Mitochondrial diseases are a group of genetic disorders with high heterogeneous that are characterized by dysfunctional mitochondria [1]. The minimum prevalence of all mitochondrial diseases is 1:5000 [2] and the clinical manifestations may present at any age [3]. Most of the mitochondrial disease patients harbor a defect in the oxidative phosphorylation system (OXPHS) which is the core and very important step for energy production [4].

*TARS2* (*MIM 612,805) gene was first described by Bonnefond and mapped the gene to chromosome 1 in 2005 [5]. The gene encodes a mitochondrial aminoacyl-tRNA synthetase, a group of ubiquitous ‘house-keeping’ enzymes that perform an integral step in the initiation of translation by charging tRNAs with their cognate amino acids [6]. The enzymes are connected to many kinds of specific diseases including cancer, neuronal pathologies, autoimmune disorder, and disrupted metabolic conditions [7]. Many disease-causing mutations have been reported [8–11]. The first and the only report connecting *TARS2* with the disease was published in 2014. Diodato et al. identified two compound heterozygous mutations in 2 siblings by whole-exome sequencing (WES) who presented with axial hypertonia, limb hypertonia, psychomotor delay, and increased serum lactate. Both of them died a few months after birth because of a metabolic crisis [12].

Nowadays, next-generation sequencing (NGS) or massively parallel sequencing has emerged as a powerful tool for testing genetically heterogeneous conditions including mitochondrial disorders [13–16]. The Mitochondrial Medicine Society demonstrated that this technology should be the preferred methodology when testing mtDNA and should be performed when mitochondrial disease is suspected [17]. Here we presented a patient with combined oxidative phosphorylation deficiency 21 who was diagnosed through clinical and molecular investigation.

## Case presentation

The proband was a 4-month-old boy at the time of admission. He was born at full-term in an uneventful pregnancy. His birth weight, height, and head circumference were 2200 g (3^th^ percentile), 48 cm (15^th^ percentile), 33 cm (5^th^ percentile) respectively. He had no so much cry after birth and was first referred to the local hospital because of afebrile convulsion about four times a day 1 month before he came to our hospital. The seizures often lasted for one minute and were characterized by staring, motion arrest, cyanosis, and rigidity of the extremities. The lactic acid level was elevated above the normal level (8.7 mmol/L, normal range < 2.0 mmol/L) and no obvious abnormalities were found in the routine examination for cerebrospinal fluid (CSF) and blood. The seizure wasn’t controlled with valproate (VPA, 21 mg/kg/d) and his parents stopped the treatment on their own after discharge. He had a fever 20 days ago. The seizures were more frequent than before accompanied by poor mental response and the lactic acid level was still higher than normal level (3.2 mmol/L). After cephalosporin treatment in the local hospital, his temperature returned to normal, but seizures were not relieved and his mental reaction was still poor.

**Fig. 1:**
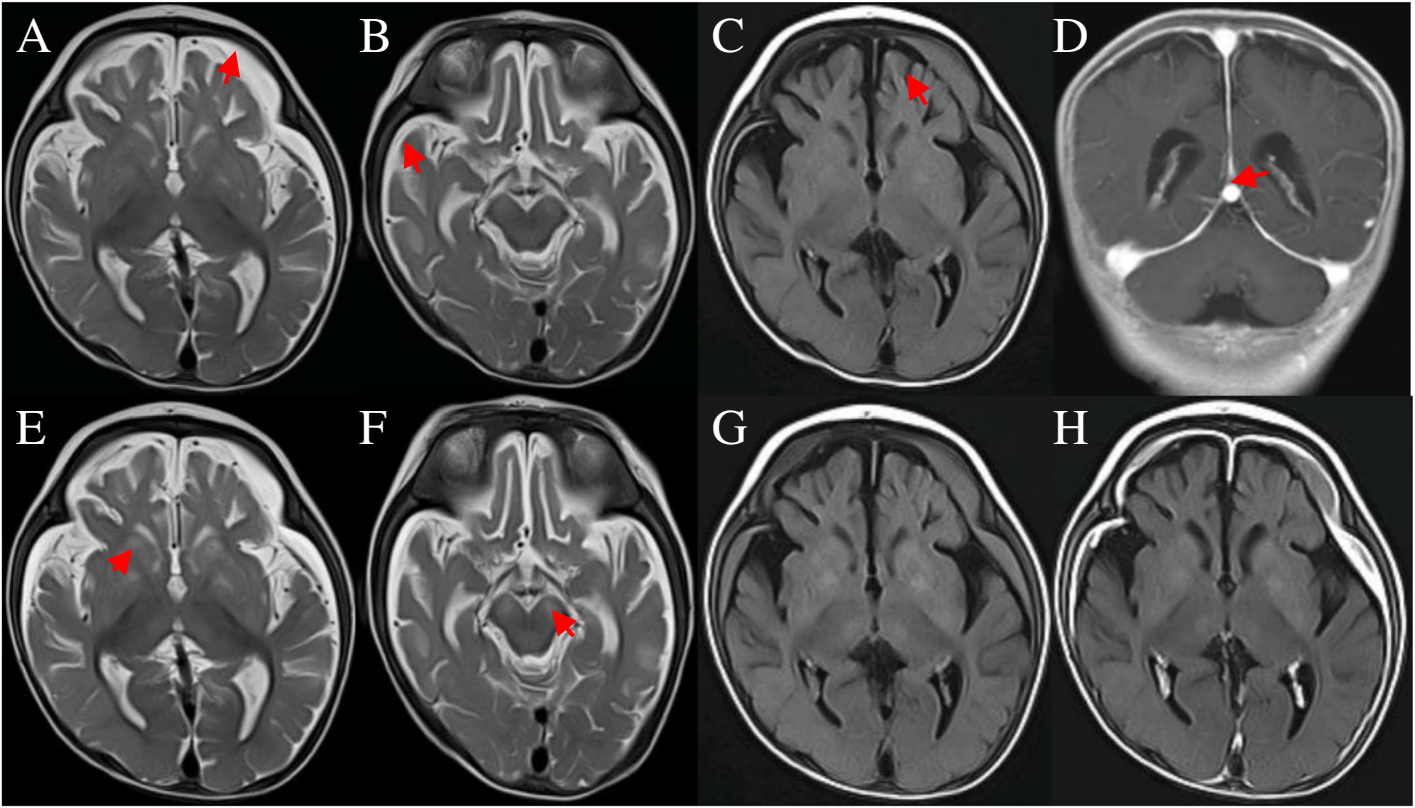
3T Brain MRI of the patient: **a** to **d** were performed when he was first transfer to our hospital at 4-month-old. **a** and **b**: axial T2WI showed arc-shaped subdural empyema was seen in bilateral frontotemporal parietal lobes. **c**: axial T2 Flair showed arc-shaped subdural empyema and bilateral cerebral hemisphere atrophy. **d**: Coronal T1WI enhanced scanning showed bilateral frontotemporal parietal dura enhanced which was called Mercedes-Benz sign. **e** to **h** was reexamined 7 days after hospitalization. **e** and **f**: axial T2WI showed new symmetrical hypersignal lesions in bilateral basal ganglia, thalamus and cerebral peduncle. **g**: the lesions had a slightly higher signal on T2Flair. **h**: the lesions had no obvious enhancement on T2Flair

**Fig. 2:**
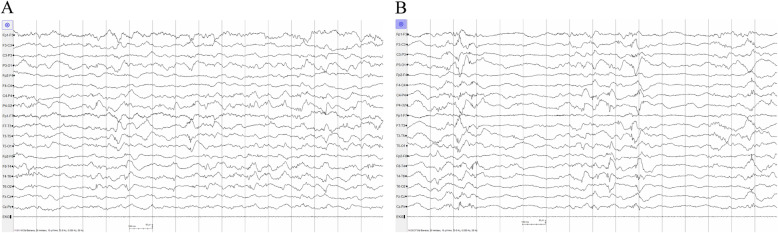
Electroencephalogram: **a**: abnormal background activity with diffuse low and moderate amplitude slow activity of 1.5-2 Hz. **b** the posterior head was dominated by frequent multi-focal sharp wave, spike wave, and slow spike wave, with medium voltage suppression of 1–2 s

He was then referred to our hospital. The global development delay with the parameters including the head circumference: 38 cm (3^rd^ percentile), body length: 56 cm (3^rd^ percentile), and weight: 5.1 kg (5^th^ percentile) were revealed. His vital signs were still stable, although he had poor eye-eye contact and poor responses, and increased muscle tone of the limbs. The lactic acid level remained high (3.6 mmol/L) at initial admission, while CSF with mild polycytosis (white blood cell count = 30 × 10^6^/L), mild increased protein level (0.66/L), normal glucose and chloride levels were detected. The CSF cultures were negative. Blood routine test, C-reactive protein and organs function were generally normal. Blood procalcitonin was 0.845 ng/ml, and sputum culture was positive for klebsiella pneumoniae. Plasma amino acid analysis showed an increase in alanine and proline content, indicating a metabolic disorder of lactate-pyruvate. Analysis of acyl-carnitine showed a significant increase in C2 and C4-OH, suggesting ketosis. The brain magnetic resonance imaging (MRI) showed an atrophy-like changes in the bilateral hemisphere and subdural effusion in the frontotemporal (Fig. 1). Electroencephalogram showed abnormal background activity with diffuse low and moderate amplitude slow activity of 1.5–2 Hz. The posterior head was dominated by frequent multi-focal sharp wave, spike wave, and slow spike wave, with medium voltage suppression of 1–2 s (Fig. 2). Brainstem auditory evoked potential revealed no obvious differentiation of bilateral I, III and V waves and bilateral auditory response thresholds were not differentiated. Echocardiography showed left to right shunt in the horizontal chamber, with a diameter about 3.5 mm. The initial diagnosis was suspected bacterial meningoencephalitis, epilepsy and psychomotor delay. After antibiotic therapy and anti-epileptic treatment (topiramate), the baby still had frequent seizures. Repeated examination of brain MRI showed abnormal symmetry signals in bilateral basal ganglia, thalamus and pedunculus cerebri. There was no significant changes in bilateral hemispheric atrophy and subdural effusion (Fig. 1). Mitochondrial energy metabolism disorders were considered and cocktail therapy was administrated including intravenous L-carnionc and oral coenzyme Q10, folic acid, vitamin B1, B2 and E. Antiepileptic treatment with levetiracetam was then added. The seizure reduced significantly and mental reaction improved. He was well fed and then discharged. Two months after discharge, he was readmitted to our hospital for poor mental response, during which he developed recurrent apnea with cyanosis and the heart rate below 60 beats/min. Despite adequate treatment of cocktail therapy, the apnea became progressively worse, and eventually the parents gave up the further treatment. The patient was followed up for 6 months after discharge and only levetiracetam was used during this period. At the last follow-up, he still had frequent seizures, and could not eat independently and remained restricted growth and development.

Genomic DNA was extracted from whole blood using QIAamp Blood Mini Kit (Qiagen, Hilden, Germany) and then sequenced on Illumina HiSeq X Ten platform (Illumina, Inc., San Diego, CA, USA). The reads were analyzed and annotated by an in-house pipeline. According to the biological significance of the mutation site, combined with the results of HGMD, ClinVar disease database and various protein function prediction software for analysis, priority is given to candidate variables related to disease phenotype. The putative mutation was further confirmed by Sanger DNA sequencing. All variant pathogenicity assessments are based on the joint consensus recommendation of the American College of Medical Genetics and Genomics (ACMG) and the Association for Molecular Pathology [18]. For copy number variants (CNV) analysis, we focused on the ultra-rare ones which were not been recorded in the Database of Genomic Variants (DGV) and the size which beyond 500 kb.

**Fig. 3:**
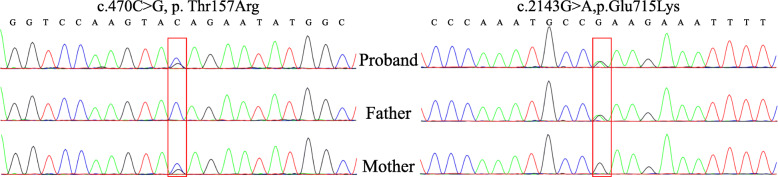
The compound heterozygous variants were detected by whole-genome sequencing and confirmed by Sanger sequencing

**Fig. 4:**
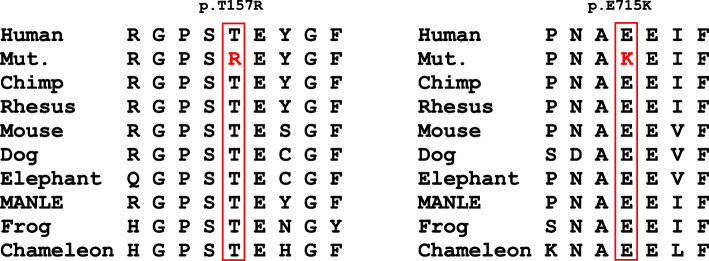
The variants region is conserved among human, Chimp, Rhesus, Mouse, Dog, Elephant, MANLE, Frog, and Chameleon. Mut., mutant; MANLE, Mandrillus leucophaeus

Finally, together with inheritance model analysis, two promising variants in *TARS2* (c.470C > G and c.2143G > A) that were inherited from their parents, respectively, draw our attention after strict filtering. Also, the two variants were further validated by Sanger sequencing (Fig. 3). These two variants have not been reported in Pubmed, nor have they been cited by HGMD and ClinVar database. The minor allele frequency (MAF) of these two mutations in the gnomAD, ExAC and 1000 Genomes database is very low or non-existent and multiple lines of computational evidence support a deleterious effect on the gene or gene product (Table 1). The two residues are conserved among species, including humans, Chimp, Rhesus, Mouse, Dog, and Elephant respectively (Fig. 4). These two variants were interpreted as likely pathogenic based on ACMG guidelines (PM1, PM2, PP1 and PP4).

**Table 1:** The analysis of pathogenicity of the variants in *TARS2*

Gene	Transcript	Mutation	Variant origin	MAF			CADD	GERP + +	SIFT	PolyPhen	Mutation Taster	PROVEAN
				gnomAD (EAS AC)	ExAC (EAS AC)	1000 Genomes						
*TARS2*	NM_025150.4	c.470C > G p.Thr157Arg	paternal	0.000163	0.000116	NE	22.6	5.23	D	PD	D	D
*TARS2*	NM_025150.4	c.2143G > A p.Glu715Lys	maternal	NE	NE	NE	25.7	5.25	D	PD	D	D

*MAF* minor allele frequency, *NE* not exist, *D* disease causing or deleterious or damaging, *PD* possibly damaging, *EAS AC* East Asian ExAC

## Discussion and Conclusions

COXPD-21(Phenotype MIM number: 615918) is rare mitochondrial encephalomyopathy caused by mutation of the *TARS2* gene on chromosome 1q21.2. The first pedigree was reported in 2014 by Diodato. They identified compound heterozygous variants c.845C > T p. Pro282Leu and c.695 + 3A > G in two siblings with early-onset mitochondrial encephalopathies by whole-exome sequencing. The two variants segregated within the family and the amount of *TARS2* proteins and threonyl-tRNA levels was decreased in samples of afflicted patients according to the genetic defect. Wang et al. further analyzed the mechanism of the p.Pro282Leu mutation and clarified the molecular consequence of the mutation [19]. They found that the mutation induced both structural and functional defects by vitro and vivo analyses.

A PubMed search for reported cases of *TARS2* and COXPD-21 yield no result but we find one abstract through Google Scholar search. Lucas Bartl et al. (2018) reported one pediatric patient with a putative splice-site mutation and missense mutation in *TARS2* who present with hypotonia, cerebellar atrophy, psychomotor delay, and increased blood-lactate underwent whole-exome sequencing [20]. Both the two variants are predicted to cause loss of mtThrRS function and the author demonstrated that the two mutations cause mitochondrial dysfunction and are therefore causal for the disorder. Unfortunately, there were no further details about the two variants in this abstract.

*TARS2* gene encodes a 718 length protein that belongs to mitochondrial aminoacyl-tRNA syntheses. This protein family has been associated with diverse clinical presentations such as hypertrophic cardiomyopathy, leukoencephalopathy with brain stem, and spinal cord involvement and lactate elevation(LBSL), Pontocerebellar hypoplasia type 6, progressive sensorineural hearing loss and ovarian dysgenesis and Charcot-Marie-Tooth disease [9, 10, 21–24]. As reviewed by González-Serrano et al., the majority of clinical manifestations are in the central nervous system [25]. Unfortunately, the pathogenic mechanism is not fairly well understood.

Here we described one infant suspected with congenital metabolic disease. By WGS analysis, we identified two putative variants in *TARS2* and no other promising variant was identified. These two variants were novel and classified as likely pathogenic according to ACMG guidelines. Certainly, further study, such as measuring the enzyme activity of mitochondrial respiratory chain complex, is required to understand functional and structural changes of these two variants. Including our case, until now, there were 4 patients with TARS2 mutations reported, and they are summarized in Table 2.

**Table 2:** Cases with *TARS2* mutations including reported and the case in our study

Pedigree	patient	Clincal phenotype	Pathogenic mutation	Reference
Family 1	Two siblings (Female/Male)	Axial hypotonia, limb hypertonia, psychomotor delay, high levels of blood lactate, both died a few months after birth of a metabolic crisis	NM_025150.4:c.845C> T(p.Pro282Leu), Maternal / NM_025150.4:c.695 + 3A > G, Paternal	[12]
Family 2	Not available	Hypotonia, cerebellar atrophy, psychomotor delay, and increased blood‐lactate	Not available	[20]
Family 3	Male	Limb hypertonia, epilepsy, psychomotor delay, and high levels of blood lactate	NM_025150.4:c.470C > (p. Thr157Arg), Maternal / c.2143G > A p. Glu715Lys, Paternal	[This Study]

In summary, this is the fourth case with COXPD-21 disease worldwide and the first in China which was diagnosed by a clinical and molecular investigation. We expand the mutation spectrum of combined oxidative phosphorylation deficiency. This contributes to the understanding of the connections between tRNA synthetase function and health. Besides, this study also suggests that WGS is a powerful technology that permits researchers to study the disease with highly phenotypic heterogeneity and will become more widely used in clinical settings.

## Data Availability

Sanger sequencing data of the variant in *TARS2* was deposited in the DDBJ database (Project ID: 5f7de96aa3c8820eeb82a9df). Human reference genome used in this study (UCSC hg19) can be downloaded at the following url: https://hgdownload.soe.ucsc.edu/downloads.html. All other data and materials mentioned in this manuscript, except patient’s private information, can be promptly available to readers by a reasonable request to the corresponding author.

## Abbreviations

WGS: Whole-genome sequencing
NGS: Next-generation sequencing
CSF: Cerebrospinal fluid
MRI: Magnetic resonance imaging
ACMG: American College of Medical Genetics and Genomics
HGMD: The Human Gene Mutation Database
MAF: Minor allele frequency
MIM: Mendelian Inheritance in Man
